# An Application of Imipenem Discs or *P. aeruginosa* ATCC 27853 Reference Strain Increases Sensitivity of Carbapenem Inactivation Method for Non-Fermenting Gram-Negative Bacteria

**DOI:** 10.3390/antibiotics10070875

**Published:** 2021-07-19

**Authors:** Tomasz Bogiel, Mateusz Rzepka, Eugenia Gospodarek-Komkowska

**Affiliations:** Microbiology Department, Ludwik Rydygier Collegium Medicum in Bydgoszcz, Nicolaus Copernicus University in Torun, 9 Maria Skłodowska-Curie St, 85-094 Bydgoszcz, Poland; mateusz.rzepka@cm.umk.pl (M.R.); gospodareke@cm.umk.pl (E.G.-K.)

**Keywords:** *Acinetobacter baumannii*, carbapenemases, carbapenemase detection, carbapenems, CIM, Gram-negative rods, imipenem, non-fermenting rods, *Pseudomonas aeruginosa*

## Abstract

Non-fermenting Gram-negative rods are one of the most commonly isolated bacteria from human infections. These microorganisms are typically opportunistic pathogens that pose a serious threat to public health due to possibility of transmission in the human population. Resistance to beta-lactams, due to carbapenemases synthesis, is one of the most important antimicrobial resistance mechanisms amongst them. The aim of this study was to evaluate the usefulness of the Carbapenem Inactivation Method (CIM), and its modifications, for the detection of carbapenemase activity amongst non-fermenting Gram-negative rods. This research involved 81 strains of Gram-negative rods. Of the tested strains, 55 (67.9%) synthesized carbapenemases. For non-fermenting rods, 100% sensitivity and specificity was obtained in the version of the CIM test using imipenem discs and *E. coli* ATCC 25922 strain. The CIM test allows for differentiation of carbapenems resistance mechanisms resulting from carbapenemase synthesis from other resistance types. It is a reliable diagnostic method for the detection of carbapenemase activity amongst non-fermenting Gram-negative rods. Application of imipenem discs and *P. aeruginosa* ATCC 27853 reference strain increases CIM results sensitivity, while imipenem discs and *E. coli* ATCC 25922 strain use maintains full precision of the test for non-fermenting rods.

## 1. Introduction

Non-fermenting Gram-negative rods are one of the most commonly isolated bacteria from human infections, especially in immunocompromised individuals. Their natural resistance to antimicrobials and relatively low nutritional requirements classify them as some of the most dangerous hospital pathogens. In addition, these bacteria have a possibility to exchange genetic material, including those encoding acquired antimicrobial resistance mechanisms [1,2]. One of the mentioned mechanisms may be resistance to carbapenems, which are often the last-chance drugs in the treatment of infections caused by Gram-negative rods, both for members of the Enterobacterales order and non-fermenters. Resistance to carbapenems is most often of enzymatic nature (synthesis of carbapenemases—enzymes hydrolyzing a number of beta-lactam group drugs). Due to the possibility of horizontal and vertical transmission of carbapenemases coding genes, it is necessary to effectively detect bacterial strains with this drug-resistance phenotype [3,4,5]. It is of great epidemiological importance in terms of both limiting the frequency of infection as well as these strains spreading in the environment. However, the available diagnostic methods for detecting carbapenemase activity amongst Gram-negative rods differ significantly in their sensitivity and specificity.

Many methods are currently used to detect carbapenemase amongst Gram-negative rods [4,6]. Chromogenic media may be used to culture and relatively quickly identify strains of carbapenem-resistant bacteria directly from a clinical specimen. It is a screening method based on the ability of bacteria to grow on a selective medium with the addition of an antibiotic [7]. Carba NP and CarbAcineto biochemical tests are other relatively fast methods [8,9]. Their principle is based on the hydrolysis of imipenem by a lysate of carbapenemase-producing bacteria. A positive result decreases pH in the reaction medium, and changes the indicator color (phenol red) [10,11]. The European Committee on Antimicrobial Susceptibility Testing (EUCAST) also recommends the use of specific tests based on the disc diffusion method: ethylenediaminetetraacetic acid (EDTA) test for metallo-beta-lactamases, boronic acid test for KPC (*Klebsiella pneumoniae* Carbapenemases) and temocillin test for OXA-48-like enzymes. However, the last mentioned test shows low specificity for OXA-48 type carbapenemases [6]. It also fails to detect oxacillinases, which are often produced by non-fermenting rods. For this reason, synthesis of these enzymes should be confirmed by other available methods [6]. This involves methods such as mass spectrometry and the Carbapenem Inactivation Method (CIM), developed in 2015 and applied to detect carbapenemases exclusively [6,12].

The aim of this study was to: (1) evaluate CIM test usefulness for the detection of carbapenemases activity amongst non-fermenting Gram-negative rods using primary test version—meropenem discs and *Escherichia coli* ATCC 25922 strain; (2) evaluate usefulness of CIM test modifications—using imipenem discs and/or *Pseudomonas aeruginosa* ATCC 27853 strain; (3) compare the results of the applied CIM versions.

## 2. Materials and Methods

### 2.1. Bacterial Isolates

In this study, 81 strains of Gram-negative rods were used. This selection included isolates obtained from the Microbiology Department of the Ludwik Rydygier Collegium Medicum in Bydgoszcz Nicolaus Copernicus University in Torun (Poland) collection and reference strain (Table S1). Based on the results obtained by the MALDI-TOF MS method (Matrix-Assisted Laser Desorption/Ionization Time-of-Flight Mass Spectrometry) (MALDI-Biotyper, Bruker Daltonik, Germany), the strains were identified and subsequently divided into two species: *Acinetobacter baumannii* (*n* = 44, 54.3%) and *P. aeruginosa* (*n* = 37, 45.7%) (Table 1 and Table 2). They were isolated from various patients, different clinical specimens, from cases of infections or colonization (Table S1). The susceptibility of the tested strains was previously determined during the diagnostic procedures: 76 (93.8%) strains were resistant to at least one of the carbapenems (imipenem or meropenem), while 5 (6.2%) strains were sensitive or intermediate to the mentioned beta-lactams. The interpretation of the susceptibility determination of the strains was made from the document “European Committee on Antimicrobial Susceptibility Testing, Breakpoint tables for interpretation of MICs and zone diameters, Version 7.1, valid from 10 March 2017” [13].

### 2.2. Analysis of Carbapenemase-Positive Gram-Negative Rods

The synthesis or absence of carbapenemases among the tested strains have been previously confirmed during standard diagnostic procedures and using additional molecular studies (Table S2). For this purpose, the following methods were used: Carba NP/CarbAcineto, disc-diffusion method (EDTA test), BD Phoenix NMIC-502 panels (Becton Dickinson) and genetic methods based on real-time polymerase chain reaction, IVD tests to detect carbapenemases encoding genes: VIM-, IMP-, KPC-, NDM-type, OXA-48 in CPE BD MAX Assay, Becton Dickinson or KPC-, VIM-type, NDM-1, OXA-48, OXA-181, CTX-M1, CTX-M9 of eazyplex SuperBug CRE test, Amplex Diagnostics. Of the 81 strains tested, 55 (67.9%) were carbapenemase-positive. All of the tested strains have been characterized for the presence or absence of carbapenemase synthesis by at least two methods (phenotypic and/or molecular), performed independently and obtaining compatible results (Table S2).

### 2.3. Carbapenem Inactivation Method Procedure

To perform CIM, the tested and reference strains were plated on Mueller–Hinton agar (MHA, *bio*Mérieux) and incubated for 16 h at 37 °C.

The method was performed in the primary version using a meropenem disc (10 µg) with an *E. coli* ATCC 25922 reference strain (A), and its three further modifications: (B) meropenem disc (10 µg) and *P. aeruginosa* ATCC 27853 strain, (C) imipenem disc (10 µg) with *E. coli* ATCC 25922 strain and (D) imipenem disc (10 µg) and *P. aeruginosa* ATCC 27853 strain; antibiotic discs were purchased from Becton Dickinson.

**Figure 1:**
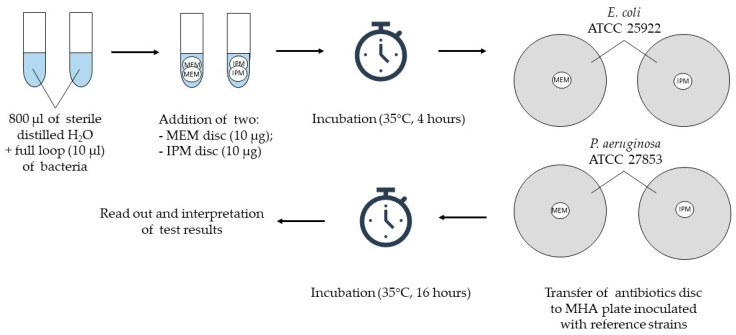
Project design of the primary version of CIM and its modification; ATCC—American Type Culture Collection, IPM—imipenem, MEM—meropenem, MHA—Mueller–Hinton agar.

To detect carbapenemase activity, suspensions of the tested strain were prepared simultaneously in two 1.5 mL test tubes (Eppendorf) by adding 800 µL of sterile distilled water and two inoculation loops (approximately 10 µL) of bacteria. Then, two meropenem discs were added to the first tube and two imipenem discs to the second one. The suspensions, together with antibiotic discs, were incubated for 4 h at 35 °C in a thermoblock (Thermomixer comfort; Eppendorf) with a mixing function (500 rpm). At the end of the incubation, two suspensions of the reference strains (*E. coli* ATCC 25922 and *P. aeruginosa* ATCC 27853) with a density of 0.5 of the McFarland scale were prepared in 0.9% sodium chloride (Polpharma). Subsequently, the suspensions were inoculated onto MHA using a disposable swab (Figure 1). Both mentioned strains are characterized by sensitivity to carbapenems, and were used in this study as indicator strains.

After the incubation step, meropenem discs were removed from the suspensions of the tested strain and placed on an MHA plate inoculated with *E. coli* ATCC 25922 and *P. aeruginosa* ATCC 27853 strains, respectively. Imipenem discs were handled in the same manner. Incubation was carried out at 35 °C for 16 h, and the results were read out and interpreted afterwards.

### 2.4. Interpretation Criteria for the Obtained CIM Results

After the incubation step, the diameters of the growth inhibition zones of the reference strains around meropenem and imipenem discs were measured. Results interpretation criteria for the tested strains were based on the analysis of the results obtained for the control strains, as well as literature recommendations [14]. The results were classified as positive, ambiguous or negative when the diameters of the inhibition zone of *E. coli* ATCC 25922 strain were as follows: 15 mm or less, 16 mm–18 mm or 19 mm and more. The corresponding values for *P. aeruginosa* ATCC 27853 strain were: below or 12 mm, 13 mm–15 mm, and more than 15 mm.

**Figure 2:**
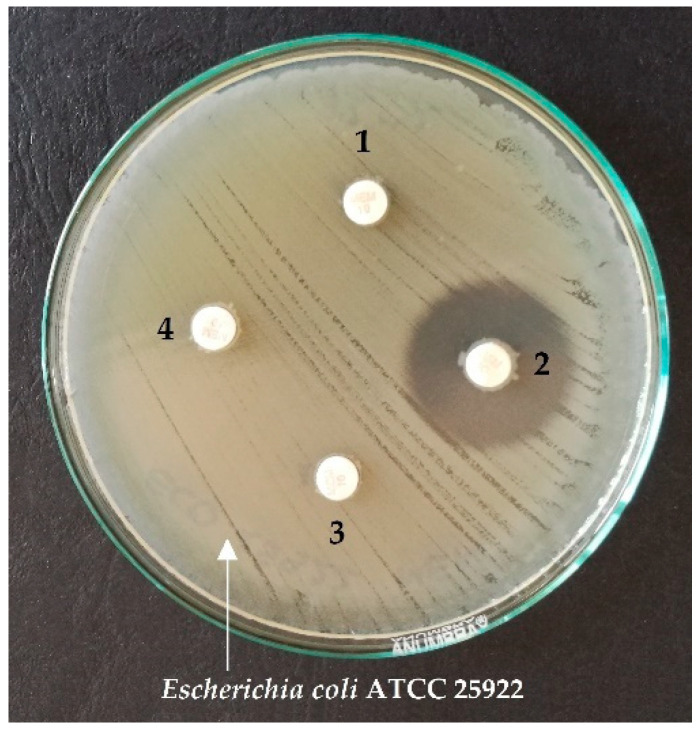
An example of CIM test results using meropenem discs and the reference strain *E. coli* ATCC 25922 for the chosen strains 1, 3, 4—positive results; 2—negative result.

Figure 2 shows an example of CIM test results for positive and negative controls, and two strains tested on the MHA plate, with *E. coli* ATCC 25922 strain culture and meropenem discs.

Ambiguous results were classified as false negative when the tested strain was positive for carbapenemases or their genes. For the tested strains that did not synthesize carbapenemase or were negative for the carbapenemase gene, equivocal results were counted as false positive.

A narrow ring of growth around the discs resulting from the carryover of the tested bacteria in the water was ignored, according to the Clinical and Laboratory Standards Institute Guidelines and the recommendations of other researchers [14,15].

### 2.5. Statistical Analysis

The sensitivity and specificity of the tests, as well as 95% confidence intervals (CI), were calculated using PQStat for Windows, version 1.8.2 (PQStat Software, Poznan, Poland). Descriptive statistics were given as number and percentage for categorical variables.

## 3. Results

### 3.1. CIM Test Results

In variant A of the CIM test, using a meropenem disc and *E. coli* ATCC 25922 reference strain, positive results were obtained for 48 (59.3%) strains and negative results for 31 (38.3%) strains. Carbapenemases were not detected in three *A. baumannii* and two *P. aeruginosa* strains, giving false negative results. Ambiguous results were obtained for two *A. baumannii* strains (Table 1). For the bacteria previously found to be carbapenemase gene-positive, the CIM test was repeated to eliminate random laboratory mistakes and the results obtained initially and secondly were consistent.

In the modified version (B) of the CIM test, using a meropenem disc and *P. aeruginosa* ATCC 27853 reference strain, positive results were obtained for 57 (70.4%). Negative results in the CIM test were found in 23 (28.4%) carbapenemases-negative strains. Among the carbapenemase-negative strains, two false positive results were obtained (*A. baumannii* and *P. aeruginosa*), while for one *P. aeruginosa* strain the result was determined as equivocal (Table 2).

In variant C of the CIM test, with the replacement of the meropenem disc with an imipenem disc, positive results were obtained for 55 (67.9%) and negative results for 26 (32.1%) strains. No false results were found (Table 2).

Table 1 and Table 2 also present the results of the CIM test in modification D, using the imipenem disc and *P. aeruginosa* ATCC 27853 reference strain. Positive results were obtained for 57 (70.4%) strains, two of which were false positives (*P. aeruginosa* and *A. baumannii*). Negative results of the CIM test were obtained for 4 (4.9%) strains. Of the carbapenemase-negative strains, 20 (24.7%) showed ambiguous results.

### 3.2. Evaluation of CIM Test Parameters in Its Different Variants

The use of CIM test modifications resulted in an increase in the diagnostic sensitivity of the test. For non-fermenting rods, only the use of imipenem discs and *E. coli* ATCC 25922 strain (variant C) in the CIM test resulted in 100% values of both parameters (sensitivity and specificity) used to assess the usefulness of the diagnostic test (Table 3).

## 4. Discussion

A spread of Gram-negative rod strains, resistant to antimicrobials, especially those producing carbapenemases, is a global threat to the effectiveness of antibiotic therapy [16,17,18,19]. This phenomenon can be a particular problem for health care units, in which carbapenems are commonly used for the treatment. The prevalence of such strains underlines the need to establish and apply rapid and reliable methods for carbapenemases detection.

Various methods are recommended, and widely used, to detect carbapenem resistance [4,6]. One of these is the CIM test, as of typical functional and enzyme synthesis-depending methods, evaluated in this paper. It distinguishes types of bacterial resistance to carbapenems resulting from carbapenemases synthesis from the resistance caused by other mechanisms, e.g., efflux pump—an active antibiotic elimination from bacterial cells [12]. Presence of the latter mentioned mechanism of resistance is of high relevance, particularly in the carbapenem-resistant phenotype in non-fermenting Gram-negative rods (especially *P. aeruginosa* strains). Its presence, additionally, explains the existence of uncommon susceptibility phenotypes, e.g., resistant to carbapenems with remaining sensitivity to chosen cephalosporins [20,21,22].

In this present work, the diagnostic value of the primary version of the CIM test and its three modified variants was evaluated.

Many studies [12,23,24,25,26] have demonstrated the high sensitivity and diagnostic specificity of the CIM test in the detection of carbapenemases produced by Enterobacterales rods. However, few studies have evaluated this test for non-fermenting rods. In one study, conducted by Madkour et al. [27], simultaneously for Enterobacterales and non-fermenting rods strains, CIM diagnostic sensitivity of 95.7% and a specificity of 95.5% were observed. The differences observed in the diagnostic sensitivity and specificity of the cited authors may result from different species characteristics, particular strain properties, and the number of the strains included in the study itself.

There are only a few reports in the available literature in which diagnostic parameters of the CIM test for non-fermenting rods were evaluated. In this paper, in variant A of CIM (meropenem + *E. coli*), false negative results were obtained for three *A. baumannii* and two *P. aeruginosa* strains. In addition, for two *A. baumannii* isolates, the result of CIM was classified as ambiguous. The false-negative results obtained with CIM may be due to the low level of carbapenemase-encoding gene expressions. In our own research, reduced sensitivity of results for non-fermenting rods was obtained, reaching 87.3%, while maintaining 100% specificity.

Van der Zwaluw et al. [12], in their study, also obtained two false negative results for non-fermenting rods, resulting in a diagnostic sensitivity of 98.8%. The results of research conducted by Aktaş et al. [28] confirm the results obtained in our study; diagnostic sensitivity of CIM was lowered for *A. baumannii* and *P. aeruginosa* strains and reached 90.0%. However, the values presented by the last mentioned author have been calculated for all of the tested strains, non-fermenting rods and Enterobacterales altogether.

Another aspect of the study was to assess the impact of modification of CIM on its sensitivity and diagnostic specificity. A replacement of the meropenem disc with an imipenem disc while maintaining *E. coli* ATCC 25922 reference strain usage (variant C) increased the ability to detect carbapenemase activity among non-fermenting rods. The diagnostic sensitivity and specificity of this method modification reached 100%.

The results of Yamada’s [29] research suggested previously that the use of imipenem discs to perform CIM test may result in false positive results for some certain bacteria species. However, these results were obtained for Enterobacterales, and not for non-fermenting rods.

Of note, the use of a meropenem discs may result in a false-negative result in the CIM test for non-fermenting rods. This is especially important for *A. baumannii* strains that produce oxacillinases most frequently. These enzymes show low hydrolytic activity towards carbapenems.

The effectiveness of imipenem disc application was confirmed by Wan et al. [30] in the simplified Carbapenem Inactivation Method test (sCIM). The use of this carbapenem resulted in the detection of carbapenemase activity in all of the tested *A. baumannii* and *P. aeruginosa* strains, while using a meropenem disc showed negative results. Our study also showed an increased sensitivity of the test with the imipenem disc compared to the meropenem disc.

An increase in the diagnostic sensitivity and specificity of the CIM test, resulting from the use of the imipenem disc, has also been demonstrated previously by Gutiérrez et al. [31]. The sensitivity and specificity of the test for 266 *P. aeruginosa* strains was higher in this study using the imipenem disc (99.4% and 98.9%, respectively) compared to the meropenem disc (91.9% and 94.7%, respectively). The diagnostic sensitivity obtained in our research (100%) may result from a significantly lower number of *P. aeruginosa* strains included in CIM test evaluation.

The results of our study also showed an increased sensitivity of the test with the imipenem disc application compared to the meropenem disc for *A. baumannii* strains with class D carbapenemases (OXA-like enzymes). As it has been previously demonstrated, OXA-like enzymes (e.g., OXA-23) present a much higher turnover rate for imipenem than for other carbapenems, including the applied meropenem [32]. It could explain our observation on OXA-like enzyme-positive *A. baumannii* isolates.

This study also examined the effect of *P. aeruginosa* ATCC 27853 reference strain application on the CIM test (variant B and D) ability to detect carbapenemase activity. Of note, this is an innovative approach and the available literature lacks any data on such modification of the CIM test. The value of the diagnostic sensitivity of the CIM test for non-fermenting rods increases, up to 100%, when using the mentioned *P. aeruginosa* reference strain, compared to its variant using *E. coli* ATCC 25922 counterpart (87.3%). Surprisingly, the simultaneous use of the *P. aeruginosa* strain and the imipenem disc significantly reduces the diagnostic specificity of the test, while maintaining its 100% sensitivity.

In summary, the CIM test is a reliable diagnostic method for detecting carbapenemase-positive strains. In addition, the uncomplicated methodology, relatively low cost and simplicity of interpretation allow for the usage of this method in laboratories too, without specialized equipment. Thus, a number of CIM test variants have been established and applied recently in laboratory practice. Some of them present increased sensitivity of results for particular species of selective carbapenemases, including differentiation between metallo-enzymes and other carbapenemases. Some of them were introduced to shorten, simplify or automate the investigation [33]. The results of our research indicate that the modified versions of CIM significantly improved sensitivity of CIM for non-fermenting Gram-negative bacteria.

Of note, the CIM test requires continuous culture of the chosen reference strain, and the time to obtain the results is relatively long, compared to the Carba NP/CarbAcineto test. However, compared to these tests, sensitivity of results, especially for non-fermenting rods, is higher and also shows a high agreement with molecular biology-based methods [8,9,27]. This particularly refers to *A. baumannii* isolates producing OXA-type carbapenemases (Table S2). Positive results of the CIM test, in comparison to generally faster genetic methods, do not provide information on the class or family of carbapenemase detected. However, this is of secondary importance to the treatment reasons. Detection of carbapenemase activity with high sensitivity and specificity is definitely more important, and the CIM test, in primary or modified variants, for particular strain groups, is definitely characterized by such parameters.

## 5. Conclusions

A CIM test is a useful tool to detect carbapenemase activity in *Acinetobacter* spp. and *Pseudomonas* spp. representatives.

Introduction of imipenem discs to the standard CIM version enhances diagnostic sensitivity and specificity of the test for non-fermenting rods.

Replacement of *E. coli* ATCC 25922 with *P. aeruginosa* ATCC 27853 reference strain in the CIM test results in sensitivity improvement of the test and a decrease in diagnostic specificity for non-fermenting Gram-negative rods.

Application of particular modifications of the CIM test as a standard microbiological procedure in a medical diagnostic laboratory may result in a significant increase of the reliability of carbapenemase detection results.

## Figures and Tables

**Table 1:** CIM test results for the carbapenemases-positive strains (*n* = 55).

Strain	Carbapenemase	*n*	Meropenem		Imipenem	
			*E. coli* ATCC 25922	*P. aeruginosa* ATCC 27853	*E. coli* ATCC 25922	*P. aeruginosa* ATCC 27853
*A. baumannii* DSM 102930	Class B (NDM-2)	1	+	+	+	+
*A. baumannii*	Class D (OXA-23)	12	+	+	+	+
		2	−	+	+	+
		1	−/+	+	+	+
	Class D (OXA-40)	25	+	+	+	+
		1	−	+	+	+
		1	−/+	+	+	+
*P. aeruginosa*	Class B (unidentified)	4	+	+	+	+
		1	−	+	+	+
	Class B (VIM)	6	+	+	+	+
		1	−	+	+	+

−—negative result, +—positive result, −/+—equivocal result, ATCC—American Type Culture Collection, DSM—German Collection of Microorganisms and Cell Cultures, *n*—number of isolates.

**Table 2:** CIM test results for the carbapenemases-negative strains (*n* = 26).

Strain	*n*	Meropenem		Imipenem	
		*E. coli* ATCC 25922	*P. aeruginosa* ATCC 27853	*E. coli* ATCC 25922	*P. aeruginosa* ATCC 27853
*A. baumannii* DSM 30008	1	−	+	−	+
*P. aeruginosa*	4	−	−	−	−
	18	−	−	−	−/+
	1	−	−/+	−	−/+
	1	−	+	−	+
*P. aeruginosa* ATCC 27853	1	−	−	−	−/+

−—negative result, +—positive result, −/+—equivocal result, ATCC—American Type Culture Collection, DSM—German Collection of Microorganisms and Cell Cultures, *n*—number of isolates.

**Table 3:** Diagnostic value of CIM test variants for non-fermenting rods (*n* = 81).

Variant of CIM Test		Sensitivity [%]	95% CI	Specificity [%]	95% CI
A	Meropenem + *E. coli* ATCC 25922	87.3	75.5–94.7	100.0	86.8–100.0
B	Meropenem + *P. aeruginosa* ATCC 27853	100.0	93.5–100.0	88.5	69.8–97.6
C	Imipenem + *E. coli* ATCC 25922	100.0	93.5–100.0	100.0	86.8–100.0
D	Imipenem + *P. aeruginosa* ATCC 27853	100.0	93.5–100.0	15.4	4.4–34.9

ATCC—American Type Culture Collection, CI—confidence intervals.

## Data Availability

The data presented in this study are available on request from the corresponding author.

## Supplementary Materials

The following are available online at https://www.mdpi.com/article/10.3390/antibiotics10070875/s1. Table S1: the origin of clinical and reference strains used in the study; Table S2: methods used to detect the activity of carbapenemases and/or carbapenemase encoding genes in the tested and reference strains.
