# Supplementary material for: An Application of Imipenem Discs or P. aeruginosa ATCC 27853 Reference Strain Increases Sensitivity of Carbapenem Inactivation Method for Non-Fermenting Gram-Negative Bacteria
Source: Antibiotics (Basel). 2021 Jul 19;10(7):875. doi: 10.3390/antibiotics10070875 (PMC8300646; doi:10.3390/antibiotics10070875)
Supplement: Supplementary file 1 [file antibiotics-10-00875-s001.zip › antibiotics-1277796-supplementary.pdf]

Table S1. The origin of clinical and reference strains used in the study

Clinical strains			
**Specimen origin**	**Species**	***n* = 78**	**%**
RTI (*n*=34)	*A. baumannii*	23	29.5
	P. aeruginosa	11	14.1
SSTI (*n*=18)	*A. baumannii*	11	14.1
	P. aeruginosa	7	9.0
BSI (*n*=10)	*A. baumannii*	3	3.8
	P. aeruginosa	7	9.0
IAI (*n*=4)	*A. baumannii*	1	1.3
	P. aeruginosa	3	3.8
UTI (*n*=4)	*A. baumannii*	3	3.8
	P. aeruginosa	1	1.3
GTI (*n*=3)	*P. aeruginosa*	3	3.8
CNSI (*n*=1)	*A. baumannii*	1	1.3
Rest (*n*=4)	*P. aeruginosa*	4	5.1

Reference strains

**Species**	**Collection and number**	**Reference**
A. baumannii	DSM No. 102930	https://www.dsmz.de/collection/catalogue/details/culture/DSM-102930
A. baumannii	DSM No. 30008	https://www.dsmz.de/collection/catalogue/details/culture/dsm-30008
P. aeruginosa	ATCC No. 27853	https://www.atcc.org/products/27853
E. coli	ATCC No. 25922	https://www.atcc.org/products/25922

ATCC – American Type Culture Collection, BSI – blood stream infections, CNSI – central nervous system infection, DSM – German Collection of Microorganisms and Cell Cultures, GTI – gastrointestinal tract infections, IAI – intra-abdominal infection, *n* – number of strains, RTI – respiratory tract infection, SSTI – skin and soft tissue infections, UTI – urinary tract infection

Table S2. Methods used to detect the activity of carbapenemases and/or carbapenemases encoding genes in the tested and reference strains

Strain	*n*	Carba NP/ CarbAcineto	Disc-diffusion method (EDTA test)	BD Phoenix NMIC-502 panel (Becton Dickinson)	CPE BD MAX Assay (Becton Dickinson)	eazyplex SuperBug CRE (Amplex Diagnostics)	Classification of the strain
A. baumannii	15	+	NA	class D	NA	OXA-40	carbapenemase-positive
	10	–	NA	class D	NA	OXA-23	
	5	–	NA	class D	NA	OXA-40	
	4	NA	NA	class D	NA	OXA-40	
	2	+	NA	class D	NA	OXA-23	
	2	–	NA	+ (unidentified class)	NA	OXA-23	
	2	–	NA	+ (unidentified class)	NA	OXA-40	
	1	+	NA	+ (unidentified class)	NA	OXA-23	
	1	+	NA	class D	–	OXA-40	
A. baumannii DSM 102930	1	+	+	NA	NDM	NA	
A. baumannii DSM 30008	1	–	–	NA	NA	–	carbapenemase-negative
P. aeruginosa	4	+	+	class B	NA	VIM	carbapenemase-positive
	2	NA	+	+ (unidentified class)	NA	NA	
	1	NA	+	class B	NA	NA	
	1	+	+	+ (unidentified class)	NA	NA	
	1	+	+	class B	NA	NA	
	1	+	NA	+ (unidentified class)	NA	VIM	
	1	+	NA	class B	NA	VIM	
	1	+	+	+ (unidentified class)	NA	VIM	
	22	–	–	NA	–	NA	carbapenemase-negative
	2	–	–	–	–	NA	
P. aeruginosa ATCC 27853	1	–	–	NA	–	NA	

- – negative result, + – positive result, ATCC – American Type Culture Collection, DSM – German Collection of Microorganisms and Cell Cultures, *n* – number of isolates, NA – not applicable
